# Role of Gene Therapy in Pancreatic Cancer—A Review

**DOI:** 10.3390/cancers10040103

**Published:** 2018-04-03

**Authors:** Mizuho Sato-Dahlman, Keith Wirth, Masato Yamamoto

**Affiliations:** 1 Department of Surgery, University of Minnesota, Minneapolis, MN 55455, USA; satom@umn.edu (M.S.-D.); wirth129@umn.edu (K.W.); 2 Masonic Cancer Center, University of Minnesota, Minneapolis, MN 55455, USA; 3 Stem Cell Institute, University of Minnesota, Minneapolis, MN 55455, USA; 4 Surgery BTR, MMC 195, 8195F, 420 Delaware St SE, Minneapolis, MN 55455, USA

**Keywords:** pancreatic cancer, pancreatic adenocarcinoma, virotherapy, oncolytic, non-viral vector, RNA interference, siRNA

## Abstract

Mortality from pancreatic ductal adenocarcinoma (PDAC) has remained essentially unchanged for decades and its relative contribution to overall cancer death is projected to only increase in the coming years. Current treatment for PDAC includes aggressive chemotherapy and surgical resection in a limited number of patients, with median survival of optimal treatment rather dismal. Recent advances in gene therapies offer novel opportunities for treatment, even in those with locally advanced disease. In this review, we summarize emerging techniques to the design and administration of virotherapy, synthetic vectors, and gene-editing technology. Despite these promising advances, shortcomings continue to exist and here will also be highlighted those approaches to overcoming obstacles in current laboratory and clinical research.

## 1. Introduction

Pancreatic cancer is now the third leading cause of cancer-related death in the United States, with pancreatic ductal adenocarcinoma (PDAC) representing the majority of these cases [1]. Despite a steady increase in survival for most cancers over the decades, the 5-year survival of PDAC remains essentially unchanged at 8% [1]. This dismal prognosis is due to a number of factors including late presentation, aggressive tumor dissemination, and lack of effective systemic therapies. 

Surgical resection with adjuvant chemotherapy remains the mainstay of curative treatment; however, at the time of diagnosis, it is estimated that only 20% of patients are eligible for resection [2]. With the addition of adjuvant chemotherapy, median survival of resectable cases is roughly 2 years, reaching a median of 28 months in a recently published trial [3]. A number of chemotherapeutic agents, particularly in combination, have been tested in the treatment of PDAC including 5-flourouracil, gemcitabine, capecitabine, nab-paclitaxel, and FOLFIRINOX (5-FU, irinotecan, and oxiplatin) [4,5,6]. While the results of these studies are certainly promising, the survival benefits are typically in the order of months. In addition, a number of factors including patient comorbidity, postoperative complications, and rapid disease progression affect the ability for patients to complete these prescribed regimens, with utilization of adjuvant chemotherapy following pancreatectomy being as low as 50% in some populations [7]. Taking into account these outcomes following what is titled a curative resection, a paradigm shift in the treatment of PDAC has been suggested, treating this as a systemic disease from the time of diagnosis [8]. 

These data highlight the current challenges which novel therapeutics for PDAC must address: (1) Improved targeting; (2) Less side effects with improved tolerance; and (3) Treatment of PDAC as a systemic disease. A promising area of research which may provide these benefits is that of gene therapies, specifically virotherapies, novel gene vectors, gene-editing technology, and RNAi therapy, which will be explored in this review. The therapies outlined here are summarized for refrence in Table 1.

## 2. Virotherapies

Virotherapy strategies provide new options for treatment of various cancers, including pancreatic cancer. Oncolytic virotherapy is one of the most promising anti-cancer agents and it has been employed for antitumoral potency via its intratumoral amplification and its strong oncolytic effect. Among them, herpes simplex virus (T-VEC, Talimogene laherparepvec, also known as OncoVEX GM-CSF) is showing positive outcomes in clinical trial and was recently approved by the US Food and Drug Administration (FDA) for use on unresectable melanoma [17,33]. Thus, oncolytic virotherapy is becoming increasingly popular for the treatment of many different forms of cancer. 

### 2.1. Replication-Based Control Oncolytic Adenoviruses

Many groups, including our own, have used adenoviruses (Ads) as a basis for the development of oncolytic agents because of the many clinically beneficial attributes and the existing rich knowledge of the adenovirus vector system [34,35]. Adenovirus vectors are known for their high in vivo gene-delivery efficiency [36], a very desirable trait and a key requirement for antitumor effect. In contrast to enveloped viruses released from cells through budding, the lytic life cycle of Ad involves the infection, replication in, and eventual destruction of host cells [37]. Recent studies also suggest that Ads have the ability to induce autophagy in cancer cells [38]. Virus-induced autophagy correlates positively with virus replication and oncolytic cell death. This characteristic is directly exploitable for oncolysis. The Ad is genetically stable, and the virus genome does not integrate into the target cell genome, meaning there is no genotoxicity [36]. 

Conventionally, adenoviral gene therapy has been performed in a replication-deficient system to avoid the possibility of toxicity resulting from adenoviral replication. To improve the antitumor efficacy without sacrificing specificity and safety, conditionally replicative adenoviruses (CRAds) have been developed. The basic concept of CRAds as oncolytic agents is that viruses replicate in tumor cells only and the subsequent lateral spread of progeny virus to surrounding tumor cells facilitates a dramatic amplification of the therapeutic effect, leaving surrounding normal cells unharmed. To date, two types of CRAds have been designed to replicate selectively in tumor cells: mutation-based and cancer-specific promoter-based.

**Figure 1:**
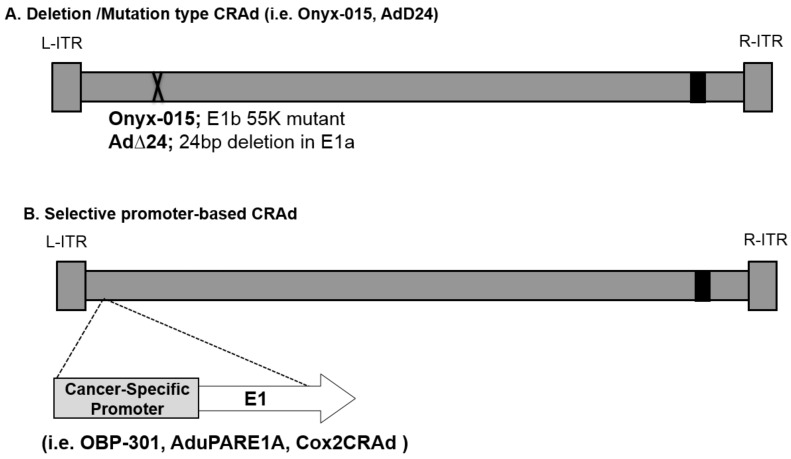
Control mechanisms of oncolytic adenovirus. (**A**) Deletion type Conditionally Replicative Adenovirus (CRAds): this type of CRAd has a mutation/deletion in a region crucial for viral replication. While cancer cells possess the cellular environment to compensate for the missing function of the virus, normal cells do not have that capability. For example, ONYX-015 (dl1520) and AdΔ24 were designed to replicate only in p53 and pRb mutated cells, respectively; (**B**) Selective promoter-based CRAd: A tumor/tissue-specific promoter controls the expression of viral genes crucial for replication. As a result, the virus can replicate only in cells in which the promoter is active. By using a promoter with a tumor-ON/normal cell-OFF profile, the replication can be restricted to cancer cells.

The first type of CRAds involved some mutations or deletion in the E1 region, which allowed replication only in specific tumors which can compensate for the loss of function due to mutation [9,39,40] (Figure 1A). The Ad mutant dl1520 (or ONYX-015) lacks the E1B region and this defect was initially expected to allow replication only in the cells with mutated p53 gene [9]. However, later studies suggested that dl1520 may not be entirely dependent upon p53 status. One of the studies has suggested that E1B is involved in cell cycle regulation and this E1B function is not essential in some cancer cells [41]. Also, Ad∆24 is another E1A-mutation-type CRAd which theoretically restricts replication to cancer cells with mutated pRb [40]. Also, we have recently generated new CRAds that are targeted to Human Papilloma Virus (HPV)-positive head and neck squamous cell carcinomas (HNSCC). These CRAds included small deletions in the E1A region of the genome (Δ24 or CB016) intended to allow for selective replication in HPV-positive cells, and they demonstrated excellent in vitro and in vivo therapeutic effects [42].

The second type of CRAds are driven by tumor-specific promoters (TSPs). This type of CRAds relies on cancer-specific, promoter-controlled transcription of the E1 region (Figure 1B). Since the E1A protein is necessary for Ad replication, promoter-controlled Ad can replicate only in cells where the controlling promoter is active. For example, OBP-301 was engineered to express E1A under the control of the human telomerase reverse transcriptase (hTERT) promoter, which is activated in various types of human cancer cells, including pancreatic cancer [10]. AduPARE1A virus drives the E1A gene under the control of the urokinase-type plasminogen activator receptor (uPAR) promoter and showed its selective replication and its strong antitumor activity in pancreatic cancer models [11,12]. Our group developed OAd controlled by cyclooxygenase-2 (Cox-2), Cox2-CRAd, for gastrointestinal cancers (e.g., pancreatic cancers [13], esophageal adenocarcinomas [43], and peritoneal dissemination of gastric cancer [44]). 

### 2.2. Enhanced Adenovirus Transduction

**Figure 2:**
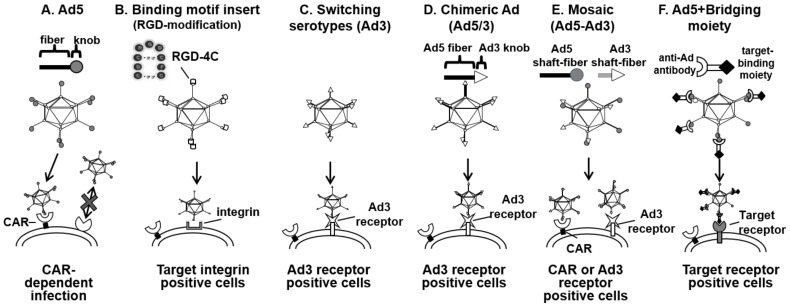
Modification of adenovirus to achieve Coxsackievirus and adenovirus receptor (CAR)-independent transduction. To achieve CAR-independent transduction, several modification strategies have been employed in adenovirus. (**A**) Poor infectivity of CAR-negative cells with conventional Ad system; (**B**) fiber modification; (**C**) switching serotypes; (**D**) chimeric; (**E**) mosaic; and (**F**) bridging molecule-based targeting.

Adenoviral infection is mediated by precise protein–protein interactions which permit the configuration of stringent transductional targeting systems, rather than lipid membrane fusion. However, the OAd infectivity in many cancers (e.g., gastrointestinal cancers, pancreatic cancers, esophageal adenocarcinomas, ovarian cancer) is extremely low due to poor expression of the adenoviral primary receptor (Coxsackie adenovirus receptor, CAR) [43,45]. Therefore, it is reasonable to develop a vector system that can transduce the target cells via another receptor. In order to solve this issue, our lab and several others have incorporated CAR-independent infection capabilities into OAd, as shown in Figure 2. Since the discovery that the “knob” domain within the Ad wild-type fiber region is responsible for CAR binding (Figure 2A), it has become a major target for infectivity enhancement. There are several ways to generate an infectivity-enhanced OAd. 

One of the most successful extrinsic binding motifs for infectivity enhancements is the incorporation of the RGD-4C motif into the HI-loop of the fiber-knob region [46,47] (Figure 2B). The RGD-4C motif is a partial peptide sequence of fibronectin identified by phage library screening [48]. Considering paucity of CAR expression in many cancer cells, effective transduction of CAR-negative cells is important for clinical usage of oncolytic adenoviruses. When it was incorporated into the HI-loop of the fiber-knob region, the Ad vector showed CAR-independent infection of the target cells. Also, OAd with this motif showed an improved cytocidal effect in CAR-negative cancer cell lines in vitro and in vivo [45,49]. 

Most Ad vectors to date are based on subtype 2 or 5. Both of them are using CAR for binding and run into the problem of poor transduction efficiency in cancer cells. Interestingly, there are other serotype Ad vectors that do not use CAR as their primary receptor. For example, Ad35 uses CD46 [50], and Ad3 uses desmogrin-2 and CD46 as its receptor for initial binding [51]. Thus, the infection of these viruses is CAR-independent. There are several more approaches for changing tropism of adenoviral vectors. One approach is to make a vector fully based on alternate subtype vectors (Figure 2C), another is to design an Ad2/5-based vector with an alternate subtype’s binding domain incorporated (chimeric or mosaic) (Figure 2D,E), and the other approach is a bridging molecule-based method, and targeting Ad by Ad library screening (Figure 2F). Switching subtype method has the advantage that all parts of the capsid consist of alternate subtype Ad proteins such as Ad3, resulting in distribution assumed to be identical to the parental virus. However, there is a risk of reduced virus replication and cytocidal effect in this approach because the other subtype’s oncolysis is not necessarily as strong as that of Ad2/5. As for the bridging molecule-based method, it can achieve the precise selectivity embodied by employing a high affinity/specificity antibody (Ab), or by using a specific binding motif for the target moiety expressed on the cell surface [52,53,54]. While promising, it is impractical to incorporate the bridging molecule into an OAd system because effective incorporation of bridging molecules into progeny viruses is not easy [13]. In recognition of this fact, chimeric fiber approaches such as Ad5/3 (Ad5 vectors with the fiber-knob domain of Ad3) are more frequently applied for OAds, and chimeric OAds displays improved gene delivery and antitumor efficacy in many preclinical studies [43,49,55,56,57]. 

Additionally, ColoAd1 (also known as enadenotucirev, EnAd), a complex and highly potent chimeric Ad3/Ad11p virus, was generated by a novel “directed evolution” approach for its ability to kill colorectal cancer cells [58]. The viral Ad11p capsid is more resistant to elimination by human serum and blood cells than Ad5 [59] which may provide an advantage for systemic delivery. ColoAd1 virus is currently undergoing several early-phase clinical trials (NCT02028442 and NCT02028117) [60].

Although incorporation of several targeting motifs has been reported to increase the infectivity of replication-deficient Ad in pancreatic cancer cells [61,62,63], the success rate of incorporating pre-identified targeting motifs into OAd has been low. To address this issue, we recently developed the high-throughput screening system using a high-diversity Ad library (>10^10^ diversity) [14]. This system employs an Ad library with seven random amino acids instead of the CAR-binding domain in the adenoviral fiber-knob region AB-loop [14]. Using this high-diversity library, we successfully isolated potent mesothelin-targeted OAd by replication-based screening [14]. Mesothelin (MSLN) is a cell surface glycoprotein that is highly expressed on pancreatic cancer, ovarian cancer, and mesothelioma [14,15]. The virus with the newly isolated MSLN-targeted OAd showed dramatic selectivity for MSLN-expressing pancreatic cancer cells in vitro and in vivo. The intravenously injected MSLN-targeted OAd showed an impressively strong antitumor effect, which was equivalent or stronger than that of intratumoral injection. Regarding systemic treatment, most systemic adenovirus therapies have been limited due to diverse factors such as liver sequestration, neutralizing antibody interactions in blood, elimination by the immune system, and physical barriers in tumors. To overcome these issues, oncolytic therapy mandates more efficient and selective gene delivery and needs to embody sufficient antitumor effect even with limited initial delivery to the tumor location. In this point, our data indicate the possibility of systemic therapy with cancer-targeted OAd by selective infection mediated. Also, Dr. Aoki’s group has constructed an adenovirus library displaying random peptides in the HI loop on the fiber knob, and identified a pancreatic cancer-targeting sequence by screening with this adenovirus library [64]. Subsequently, they generated promoter-controlled pancreatic cancer-targeted OAd that displayed the targeting sequence on the fiber knob of survivin promoter-regulated OAd (AdSur-SYE). This virus showed a much higher gene transduction efficiency and strong antitumor efficacy in pancreatic cancer with intratumoral infection [16].

In this sense, the library screening technology may have broad implications for the development of targeted gene delivery approaches.

### 2.3. Therapeutic Gene-Expressing Vector

A reasonable approach to strengthen the antitumor effect of the OAd is expressing a transgene with an antitumor effect from the oncolytic virus. This approach has been taken in a wide variety of oncolytic viruses including Ad and vaccinia [65,66]. One interesting example with Ad is interferon (IFN)-α. It has been known that IFN-α has a strong antitumor effect and has the ability to sensitize the tumoricidal effects of chemotherapeutic agents (e.g., 5-FU) and radiotherapy [67,68,69,70]. Particularly, in the field of pancreatic cancer, a multicenter phase II trial (5-FU, cisplatin, and IFN-α in conjunction with radiation therapy) confirmed the efficacy of IFN-based chemoradiation for PDAC [67]. However, despite encouraging survival results and immunological data, clinical trials have defined several problems impairing the clinical utility of IFN-α for pancreatic cancer patients: (i) Systemic toxicity of IFN-α, and (ii) Insufficient delivery and unsustainable levels of IFN-α in the tumor site due to rapid degradation of the cytokine in blood circulation and low vascularity [71,72].

In the context of IFN expression from Ad, intrinsic class I IFN expression from the infected cancer cells did not hamper Ad replication in the tumor. As a result, OAd with IFN-α showed efficient replication in pancreatic cancer cells [65,73]. In this way, OAd with IFN-α has a unique benefit for its application to pancreatic cancers. Moreover, several studies showed that the interactions between OAds and immunomodulatory molecules, such as GM-CSF and interleukin-12 (IL-12), induced an antitumor effect [74,75]. Cells of the innate immune system recognize pathogen-associated molecular patterns on the adenovirus. The production of IL-12 and GM-CSF increases, which results in activation of CD4+ and CD8+ T cells. Therefore, even if a small percentage of the cancer cells contains the target molecule for oncolytic adenoviral infection, a local pro-inflammatory response can be elicited to potentiate an antitumor response [76].

In particular, the phase III trial of T-VEC (genetically modified herpes simplex virus expressing GM-CSF) demonstrated improvements in durable response rate and a trend toward improved overall survival compared to GM-CSF alone, which led to the approval by the FDA of its use in advanced melanoma patients [17]. T-VEC is currently being tested in several other clinical trials for the treatment of pancreatic cancer, soft-tissue sarcomas, and head and neck cancer (NCT03086642, NCT03069378 and NCT02626000). 

### 2.4. Combination Therapy with Oncolytic Viruses

Combination therapies involving multiple chemotherapies and radiation have been performed in many cancers [77,78,79]. Likewise, combination therapy is possible and promising with oncolytic virus. For example, the phase II trial of reovirus (Reolysin) in combination with gemcitabine has demonstrated clinical benefit in patients with advanced PDAC (NCT00998322), with promising survival advantage and favorable toxicity profile [18]. Also, a spontaneously mutated herpes simplex virus, HF10, is currently in clinical trials for the treatment of pancreatic cancer (Phase I, NCT03252808). The repeated intratumoral injection of HF10 demonstrated that all patients tolerated the treatment well without any observed adverse effects after treatment. The response to treatment was classified as stable disease in three patients, partial response in one patient, and progressive disease in four patients [19,20]. 

Several studies have reported the combination therapy of gemcitabine and OAd. For example, oncolytic mutants lacking the anti-apoptotic E1B19K gene showed increased pancreatic cancer cell killing in combination with gemcitabine by enhancing drug-induced apoptosis [80]. Ad5/3-∆24 was used in combination with gemcitabine in ovarian cancer cells, synergistic interactions were observed that resulted in enhanced cell killing [81]. Moreover, tumor stroma-targeted OAd has been tested in combination with Gemcitabine and Abraxane^®^ (Celgene, Uxbridge, UK) in patients with pancreatic cancer and advanced solid tumors. Two phase I clinical trials with VCN-01 alone or in combination with chemotherapy are currently underway (NCT02045589, NCT02045602). These are designed to investigate the safety and tolerability of three intratumoral injections of VCN-01 (ID NCT02045589) or a single intravenous injection of VCN-(ID NCT02045602). In this study, they used hyaluronidase expressing OAd, it is named VCN-01. VCN-01 is a replication-competent adenovirus specifically engineered to replicate in tumors with a defective RB pathway (∆24), presents an enhanced infectivity through a modified fiber (RGD) and an improved distribution through the expression of a soluble hyaluronidase (PH20) [21]. 

More recently, the combination therapy of oncolytic viruses and immune-checkpoint inhibitor such as anti- CTLA-4 antibody and anti-PD-1 antibody has demonstrated promising results. For example, Ad5/3-∆24-based OAd coding for anti-CTLA4 antibody has been tested in several cancer cell lines, and a direct anti-CTLA-4-mediated pro-apoptotic effect was observed in vitro and in vivo [82]. Also, another approach which is using oncolytic viruses armed with immunostimulatory genes has been reported [22]. They generated LOAd703 which is a designed adenovirus armed with trimerized CD40L and 4-1BBL that activates the CD40 and 4-1BB pathways, respectively. Both in vitro and in vivo, the LOAd703 viruses were able to replicate and kill pancreatic cancer cells via oncolysis. A clinical trial is ongoing to investigate the safety and efficacy of repeated LOAd703 intratumoral injections combined with standard of care in patients diagnosed with pancreatic cancer not eligible for surgery (NCT02705196).

Therefore, the combination of oncolytic virus and immune-checkpoint inhibitor will be an appealing strategy. While it is promising, combinatory approach can sometimes be a double-edged sword because proper evaluation of the combination effect is not that simple: it is crucial to determine appropriate timing, dosing and sequence schedules of each agent. However, once it is established, it may make a big impact for clinical efficacy in various cancers. 

## 3. Non-Viral Gene Therapies

While the majority of novel gene therapies described in the literature and here have utilized viral vectors for delivery, non-viral technology has continued to advance with the introduction of novel lipid and polymer technology, as well as gene-editing techniques. The main advantages of non-viral vectors include the potential for less immunogenicity, larger nucleic acid payloads, and relatively easier manufacturing [83].

Barriers to overall gene therapy delivery are well described in a recent review of RNA therapeutics; these include: (1) size and charge of drug; (2) RNase/DNase susceptibility; (3) the reticuloendothelial system of the liver and kidney; (4) immunogenicity; and (5) endocytosis [84]. These challenges have been overcome in a number of interesting ways in pancreatic cancer trials including direct intratumor delivery and sustained release technology [28], various cationic and neutral lipid/liposomes carriers [85], conjugation with synthetic carriers [29], and targeting of receptors to induce endocytosis [32]. The array of potential gene products is substantial, including delivery of plasmid DNA, RNA interference technology, and gene-editing systems such as CRISPR/CAS. These applications and future directions will be reviewed in more detail here.

### 3.1. RNA Interference

**Figure 3:**
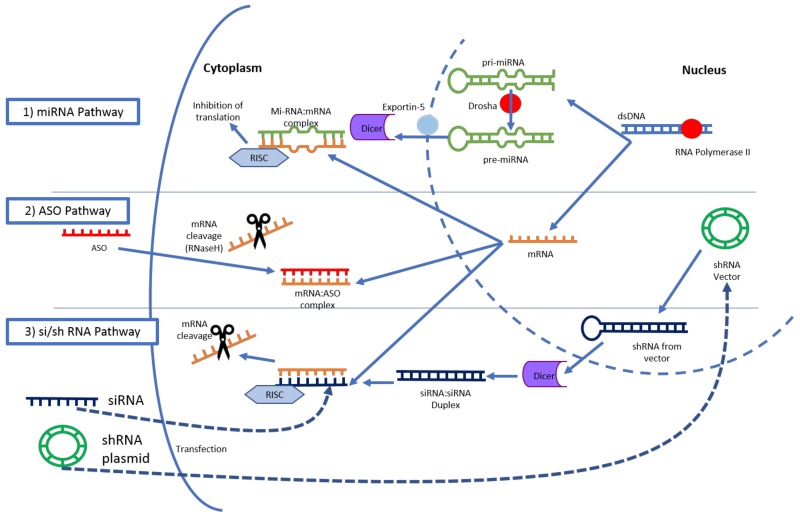
RNA Molecules and respective pathways. (1) miRNA pathway. After processing, one double-stranding miRNA associates with the RNA-induced silencing complex (RISC) complex and inhibits translational expression; (2) Antisense oligonucleotide (ASO) pathway. ASOs enter the cell cytoplasm via described mechanism, bind the target mRNA, and activate intracellular RNase enzyme; (3) siRNA pathway. dsRNA or shRNA are first altered by DICER and become mature siRNA. One strand of siRNA associates with the RISC complex and marks mRNA for degradation.

RNA interference refers to post-transcriptional gene silencing by way of miRNA, siRNA and shRNA. miRNA is of genomic origin and regulates expression of multiple RNA, and major function is translation suppression due to mismatches with the target sequences. siRNA is synthetic in origin and more targeted in effect. The effect of siRNA is considered to be both translation suppression and RNA degradation by RNaseH. shRNA is transcribed from extrinsic DNA, typically a plasmid requiring integration into the host genome [86]. siRNA are those most commonly manipulated in clinical applications due to their more simplistic sequencing and lack of genomic integration [87]. siRNA is a double-stranded RNA molecule typically of 20–30 nucleotides in length that, upon entry into the cytoplasm, is processed by DICER and then associates with the RNA-induced silencing complex (RISC). This complex then binds the complementary target mRNA which is then degraded and “silenced” [88]. (Figure 3) In contrast, antisense oligonucleotides (ASOs) represent single-stranded nucleotide sequences with synthetic modifications to the phosphodiester backbone allowing protection from degradation and enhancing cell entry [84]. ASOs do not require association with cellular machinery for degradation of mRNA [89].

One of the most attractive features of utilizing RNAi technology is the small size of payload, allowing for the possibility of large amounts of the drugs to be delivered to the target tumor. Most notable in pancreatic cancer tumor architecture, however, is its highly desmoplastic nature, with as much as 90% of tumor volume being made of stromal components [90]. In a study of polymeric micelle nanoparticle size and antitumor activity, drug penetration for pancreatic cancer was noted only in those of a particle size of 30 nm or less, in contrast to up 400 nm in the more porous colon cancer models [91]. Despite this difficulty, a number of unique approaches have been tested.

As mentioned earlier, ASOs need not associate with cellular machinery to become functional, and therefore allow for a wider range of chemical modification. ASO modifications include replacement of the nucleotide backbone with phosphothiorated, providing more nuclease stability and enhanced uptake, and 2′-*O*-methyl modification improving binding and again nuclease resistance [92]. In targeting pancreatic cancer, this has been tested clinically in ISIS-2503, and AEG35156 [23,25]. ISIS-2503 is an ASO-targeting h-ras, and important signaling molecule in PDAC implicated in tumor progression via persistent mitogenic signaling after mutation [93]. This is an example of the phosphothiorated backbone chemical medication. AEG35156 is a recently developed antisense oligonucleotide with the core DNA bases flanked by four 20-*O*-Me-modified RNA residues [25]. This drug targeted X-linked inhibitor of apoptosis (XIAP), an antiapoptotic protein which strongly inhibits caspases and is overexpressed in a number of malignancies, including PDAC [94]. Both of these ASOs did enter clinical trials, however, with disappointing results. The phase I testing of ISIS in combination with gemcitabine was well tolerated; however, it demonstrated no clinical benefit over gemcitabine alone in the phase II trial [23,24]. AEG35156 entered phase I testing, yet failed to show significant clinical activity [25]. As detailed in a recent review of ASO technology, this field is yet developing, and these early results have not yet disrupted the industry, with work on new backbones and conjugates continuing to evolve [92].

Rather than focusing on chemical modification of the sequences themselves, studies of conjugate siRNAs have shown some promising results. In addition to the addition of a liposomal conjugate, the drug ATu027 took an interesting approach to overcoming the difficult tumor penetration in PDAC. Atu027 is a siRNA-targeting protein kinase 3 (PKN3) mRNA utilizing a liposomal complex (AtuPLEX) carrier. PKN3 is a downstream effector of the PI3 kinase pathway, and interference via antisense inhibitors is thought to decrease angiogenesis and metastases via endothelial modulation [26,27]. The liposomal complex carrier includes a cationic lipid and a PEG-lipid, making it particularly effective targeting endothelial cells, rather than the PDAC tumor itself. The phase 1 trial demonstrated safety and tolerance in escalating doses delivered intravenously, with 41% of patients demonstrating disease stabilizing [85]. Of note in this trial, a wide variety of cancers were included, while the ongoing Phase II trial evaluating Atu027 (NCT01808638) is specific to advanced and metastatic pancreatic cancer.

Finally, rather than taking a systemic approach to drug delivery, administration of si-G12D-LODER in locally advanced pancreatic cancer was focused on direct intratumor delivery and sustained release [28]. This siRNA drug targeted mutant KRAS (glycine to aspartate at codon 12), an early mutation involved in over 95% of pancreatic cancer cases [95]. This intervention took advantage of a novel delivery system Local Drug EluteR (LODER-Silenseed Ltd. (Modi’in, Israel)), which is a biodegradable polymeric matrix which allows for both protection from degradation and slow stable drug release over the course of months [96]. In this phase 1/2a clinical study of si-G12D-LODER, the matrix was delivered by endoscopic ultrasound (EUS) using a biopsy needle, in a population of patients diagnosed with unresectable, locally advanced adenocarcinoma of the pancreas, or those not fit for surgery. The drug was deemed safe and well tolerated, with a median overall survival of 15.12 months. In those patient whom underwent follow-up CT scan, 10/12 demonstrated stable disease [28]. With some promising results, a randomized Phase 2b study in currently in progress (NCT01676259).

Before moving to DNA therapies, it should be noted that microRNA (miRNA) therapy is certainly being evaluated in the preclinical stages in the treatment of PDAC. miRNAs are a family of 21–25 nucleotide non-coding RNA that are typically transcribed by RNA polymerase II and act to attenuate mRNA translation [97]. A number of miRNAs have been implicated in the initiation and progression of PDAC, regulating key pathways, such as the recently targeting K-ras mutation [98,99]. Unfortunately, no miRNA therapeutics have yet entered clinical testing for treatment of PDAC, while some are indeed underway for other gastrointestinal cancers [98].

### 3.2. Plasmid DNA

Delivery of plasmid DNA (pDNA), in addition to the possible mechanisms of RNAi discussed above, offer the ability to transcribe for the replacement of mutated enzymes, suppressed signaling molecules, and for cytotoxic machinery. The notable differences in barriers to delivery of pDNA to RNAi is that of size, charge and target site of action [100]. The most common methods of overcoming these are conjugation to polyplexes such as polyethylenimine (PEI), and lipid complexes. pDNA offers the ability to target some of the key genetic alterations, including loss of tumor suppressor function, and chemoresistance [90,101].

A combination gene therapy, CYL-02, aimed at both loss of function and chemoresistance [29]. This agent targeted the somatostatin receptor subtype 2 (SSTR2), deoxycytidine kinase (DCK), and uridylate monophosphate kinase (UMK.) The SSTR2 receptor is lost in 95% of PDAC cases, and in vivo administration demonstrated significant inhibition of tumor progression [30,102]. DCK is a key enzyme in gemcitabine metabolism, and loss of expression of this enzyme has been associated with chemoresistance [103]. CYL-02 is a plasmid DNA encoding for both SSTR2 and a DCK:UMK fusion gene. In the phase 1 trial of this product, two intratumoral injections were performed via EUS with standard of care gemcitabine therapy following. This study proved the treatment safe and well tolerated; however, no significant overall survival response was found. It should be noted that the patients demonstrated two distinct responses, with some having significant improvement in progression-free survival [29]. A Phase II trial is now underway (NCT02806687).

An example of cytotoxic machinery delivery is a double-stranded DNA plasmid therapy, BC-819 (also known as DTA-H19), which has also undergone phase 1 testing [31]. BC-819 is a plasmid encoding the diphtheria toxin-A chain under the regulator of the H19 promoter, a gene which is overexpressed in malignancies and typically not transcribed postnatally [104]. Inclusion criteria biopsy specimens with positive markers for H19 expression, and no concurrent chemotherapy. The protocol included twice-weekly injections for two weeks via either EUS or a percutaneous radiographically guided approach with the plasmid and PEI transfectant. The study demonstrated BC-819 safe and an at least partial response in all full-dose patients. Interestingly, 2/9 patients receiving this treatment had tumors which responded significantly enough to deem them surgically resectable [31].

Finally, SGT-53 is a scL nanocomplex pDNA here aimed at regaining a loss of function, encapsulated in a cationic liposome [32]. The gene encoded is for the normal human wild-type p53, a commonly inactivated tumor suppressor gene in pancreatic cancer, among other malignancies, and a target for antitumor technologies [105]. Also unique to SGT-53 is “decoration” of the liposome with anti-transferrin receptor single-chain antibody fragments, allowing for both specificity and internalization in tumor cells [106]. In contrast to many vectors used to deliver this plasmid intratumorally, SGT-53 was systemically delivered via intravenous injection. This phase 1 trial was completed in a population of patients with varying solid tumors and was well tolerated. A high level of transgene expression was noted in tumor biopsies, and importantly not in normal tissue obtained. Also, 7/11 patients demonstrated stable disease, with one tumor being reclassified as operable after treatment [32]. A phase II study of combination SGT-53, gemcitabine, and nab-paclitaxel in metastatic pancreatic cancer is planned (NCT02340117).

### 3.3. Gene-Editing Technology

Recent developments in CRISPR-Cas9 gene-editing technology has been followed with significant enthusiasm for possible therapeutic applications. Without examining the mechanism in great detail, this system offers the ability to cleave desired double-stranded DNA segments from the genome by introducing CRISPR (clustered regularly interspaced short palindromic repeats) sequences marking sites for cleavage by Cas9 (CRISPR associated) protein endonucleases [107]. This surprisingly simplistic system has allowed for a number of novel approaches to understanding the natural history of PDAC and also offers potential therapeutics. Current research is mainly focusing on using this system to further investigate the mechanism of known genetic aberrations, and screen for future gene targeting and phylogenetic tracking [108,109]. Genetic disorders and oncologic trials are planned in the clinical realm with the first-in-human case report of injections of cells containing the CRISPR-Cas9 system just last year [110]. Much like the other gene technologies described above, the vectors and delivery mechanisms of this system will certainly face similar obstacles outlined here [111]. Finally, gene editing will require a much higher efficiency of transduction, as compared to oncolytic virus for example, as all aberrant cells must be edited without a significant bystander effect from cytotoxicity [112].

## 4. Conclusions

With pancreatic cancer mortality remaining unchanged over the past decades, and an expectation to surpass breast, colorectal and prostate cancer mortality by the year 2030, there is a clear impetus to develop novel therapies for the treatment of PDAC [113]. It is hoped that this review of gene therapy highlights both the promising future prospects as well as those challenges that must be overcome in the treatment of PDAC. Preclinical work in screening libraries, combination immunotherapies, and the reinvigoration of clinical trials of virotherapy are certainly encouraging. Novel compounds and nanoparticles are continuing to be developed in non-viral vector technology. Finally, the prospect of gene-editing technology to address the malignant transformation at its primary source will be eagerly awaited.

## Figures and Tables

**Table 1:** Various therapeutic proteins coupled with viral therapies.

Name	Vector/Delivery System	Route of Delivery *	References
Virus			
**ONYX-015**	Conditionally replicative adenovirus (CRAd) mutant dl1520, lacking E1B region	IV	[9]
	Mechanism: Selective replication in cancer cells with mutated p53		
**OBP-301**	CRAd—E1A-mutation type	PC	[10]
	Mechanism: Expresses E1A under the control of the human telomerase reverse transcriptase (hTERT) promoter		
**AduPARE1A**	CRAd—E1A-mutation type	IV	[11,12]
	Mechanism: Expresses E1A gene under the control of the urokinase-type plasminogen activator receptor (uPAR) promoter		
**Cox2CRAd**	CRAd—E1A-mutation type	IT	[13]
	Mechanism: OAd controlled by cyclooxygenase-2		
**MSLN-targeted OAd**	Targeted oncolytic adenovirus (OAd)	IV	[14,15]
	Mechanism: Selectivity for MSLN-expressing pancreatic cancer cells		
**AdSur-SYE**	Mechanism: Promoter-controlled pancreatic cancer-targeted OAd.	IT	[16]
	Mehcanism: Displays the targeting sequence on the fiber knob of survivin promoter		
**T-VEC**	Herpes simplex virus expressing GM-CSF	IT	[17]
	Mechanism: Sensitize the tumoricidal effects of chemotherapeutic agents (e.g., 5-FU) and radiotherapy		
**Reolysin**	Unmodified oncolytic reovirus	IV	[18]
	Mechanism: Replication in Ras-activated cancer cells, trial in combination with gemcitabine		
**HF10**	Unmodified oncolytic herpes simplex virus	IT	[19,20]
	Mechanism: Selective replication in cancer cells		
**VCN-01**	Replication-competent adenovirus	IT	[21]
	Mechanism: Selective replication in cancer cells with defective RB pathway, hyaluronidase expressing		
**LOAd703**	Immunostimulatory adenovirus, trimerized CD40L and 4-1BBL	IT	[22]
	Mechanism: Activates the CD40 and 4-1BB pathways		
RNA			
**ISIS-2503**	Antisense oligonucleotide inhibitor of H-ras	IV	[23,24]
**AEG35156**	Antisense oligonucleotide targeting X-linked inhibitor of apoptosis (XIAP)	IV	[25]
**ATu027**	siRNA targeting protein kinase 3 (PKN3) mRNA utilizing a liposomal complex (AtuPLEX) carrier	IV	[26,27]
**si-G12D-LODER**	siRNA drug targeted mutant KRAS, utilizing biodegradable polymeric matrix	IT	[28]
**DNA**			
**CYL-02**	Plasmid DNA encoding for somatostatin receptor subtype 2 (SSTR2), deoxycytidine kinase (DCK), and uridylate monophosphate kinase (UMK)	IT	[29,30]
**BC-819/DTA-H19**	Plasmid DNA encoding the diphtheria toxin-A chain under the regulator of the H19 promoter	IT	[31]
**SGT-53**	Plasmid DNA encoding normal human wild-type p53 utilizing cationic liposome carrier	IV	[32]

* IV-intravenous, IT-intratumoral, PC-preclinical, not yet tested in vivo.
